# Unshackling our youth from the chains of substance abuse as disaster risk

**DOI:** 10.3389/fpubh.2025.1643748

**Published:** 2026-01-12

**Authors:** Konosoang Sobane, Wilfred Lunga, Tshegofatso Ramaphakela, Caiphus Baloyi

**Affiliations:** 1 Human Sciences Research Council (HSRC), Pretoria, South Africa; 2 Department of Strategic Communication, The University of Johannesburg, Johannesburg, South Africa; 3 African Centre for Disaster Studies (ACDS), North-West University, South Africa; 4 University of Free State’s Disaster Management Training Education Centre (DIMTEC), Bloemfontein, South Africa; 5 Department of Social Sciences, University of KwaZulu-Natal, Durban, South Africa

**Keywords:** communication ecology, peer pressure, Sekhukhune, strategies, substance abuse, youth

## Abstract

This study, a collaboration between the University of Limpopo and the Human Sciences Research Council (HSRC), aimed to generate baseline evidence to inform the development of an innovative performative arts intervention, designed to engage young people with scientific knowledge on substance use disorders (SUD), mental health, and behavioral risks. Using focus group discussions and key informant interviews across three communities in the Sekhukhune District, data were collected from youth, community members, and traditional governance structures. The findings reveal that generational poverty, high unemployment, and the socio-economic impacts of mining investments – particularly their role in driving school dropout rates, are central drivers of youth substance abuse. Dagga, nyaope (whoonga), and cocaine emerged as the most frequently abused substances, with consequences ranging from family breakdown and crime to the erosion of social cohesion. The study concludes that substance abuse in Sekhukhune reflects both structural inequalities and weak institutional support, underscoring a multisectoral response. Evidence highlights an urgent need for school-based prevention initiatives, safe recreational alternatives, and the active engagement of faith-based and community institutions to mitigate risks and strengthen youth resilience.

## Introduction

Substance abuse among the youth in South Africa, particularly in rural regions such as Limpopo Province, remains a pressing concern, with significant implications for both mental and physical health. Research has demonstrated a strong correlation between substance abuse and various mental health issues, including cognitive and behavioral disorders, with associated consequences, such as suicide, schizophrenia, social anxiety, mood disorders like depression, and engagement in criminal activities, such as theft and violence (1). The impact of substance abuse is further compounded by the dual challenge of insufficient understanding and limited access to data on these issues, which hinders the development of effective prevention and intervention strategies.

International studies have extensively explored the relationship between substance abuse, including alcohol, illegal drugs, and prescription medication, and the onset of mental health disorders. These studies highlight a clear connection between substance use and a range of psychological conditions, including depression, anxiety, and psychosis (2). Furthermore, substance use has been linked to an increased risk of engaging in violent behavior or becoming a victim of violence. In South Africa, however, available data on substance abuse and mental health are often underutilized in shaping public health strategies and interventions targeted at the youth.

Available datasets, such as the South African National Health and Nutrition Examination Survey SANHANES-1, 2018 and the South African Community Epidemiology Network on Drug Use Human Sciences Research Council (HSRC) (79), contain valuable information regarding the prevalence of substance abuse and its association with mental health problems. However, these datasets are often not disseminated in a format that is accessible or engaging for the general public, particularly for the youth who are most affected. While these surveys highlight the high incidence of substance abuse and associated mental health issues, they fail to translate this data into actionable strategies that can empower youth, promote prevention, and encourage engagement with scientific evidence. This gap is further exacerbated by the lack of targeted interventions that prioritize youth agency, participation, and human rights within the context of substance abuse prevention and treatment.

In Limpopo Province, the prevalence of substance abuse among adolescents has been reported to be alarmingly high. Studies by Matla and Madu (3) found that 39.1% of adolescents in the Pietersburg area engaged in alcohol use, and 19.8% used illicit drugs, with the average age of initiation being approximately 15 years. A subsequent study by Govender et al. (4) revealed that the most used substances were commercially produced alcohol (54.8%), cannabis (49%), and inhalants (39%). These findings are consistent with more recent reports, including the SANCEDU (2018), which highlight high levels of substance abuse among South African youth, especially in rural areas. A 2018 field assessment by the HSRC in the Sekhukhune district further corroborated these findings, indicating persistent high rates of substance use among youth in the region.

South Africa possesses a comprehensive policy framework to address substance use disorders (SUD), anchored by the National Drug Master Plan (NDMP) 2019–2024, which emphasizes multi-sectoral coordination, prevention, harm reduction, the critical role of families, and community-based approaches (5). Key legislative instruments, including the Prevention of and Treatment for Substance Abuse Act 70 of 2008, the Integrated School Health Programme, and the National Mental Health Policy Framework, collectively support prevention, treatment, and the integration of SUD services within broader health systems (6, 7).

Despite the presence of a robust policy framework, community-level implementation of substance abuse interventions in South Africa continues to face significant challenges. Insufficient funding limited human resources, and poor interdepartmental coordination have been identified as key barriers to effective service delivery (8). Enforcement of supply reduction measures near schools and community hotspots is often weak, which undermines the effectiveness of national policies. Low levels of community engagement and limited participatory involvement further restrict local ownership and behavior change, reducing the impact of interventions. Structural factors, including poverty, unemployment, and a lack of recreational infrastructure, continue to drive substance abuse, while stigma and limited access to services impede treatment uptake, particularly in rural areas (9). Complementing the National Drug Master Plan (NDMP), sector-specific strategies align with national objectives. For example, the Department of Basic Education implements life skills and drug awareness programs in schools, while the Department of Health integrates substance abuse treatment into mental health services (10). The Prevention of and Treatment for Substance Abuse Act 70 of 2008 provides the legal framework for establishing treatment centers, early interventions, and community-based support services. However, persistent resource constraints, fragmented coordination, and limited access to services in rural areas continue to hamper effective implementation (5, 9).

Recent policy discourse increasingly advocates for youth-centered, participatory, and trauma-informed approaches to substance abuse prevention and intervention. While existing policies provide a strong foundation, bridging the gap between policy and practice necessitates enhanced resources, improved enforcement, deeper community involvement, and targeted efforts to address entrenched socio-economic determinants. Though policy and research have advanced understanding, a critical need remains for effective, accessible interventions that translate knowledge into meaningful community action and sustained behavior change. Intervention design must prioritize co-creation with youth to foster participation and empowerment (11). These initiatives should be evidence-based and leverage convergent media and multimodal dissemination strategies that resonate with young people, thereby enhancing engagement with scientific evidence and stimulating youth-led prevention efforts. While similar innovative interventions have shown promise in HIV/AIDS programming (12), comparable approaches targeting substance abuse remain limited, especially in high-burden areas like Sekhukhune, where socio-economic barriers further restrict access to evidence-based information.

## Theoretical grounding

This project is grounded in an integration of the Integrated Participatory Framework for Youth-Centred Substance Abuse Intervention (IPF), Social Cognitive Theory (SCT) (13, 14), and Narrative Therapy Theory (NTT) (15, 16). This multi-theoretical approach offers a rigorous conceptual lens for understanding how creative-arts-based interventions can simultaneously facilitate identity reconstruction and behavior change. From the SCT perspective, behavior is shaped via reciprocal interaction among personal factors, environment, and actions, with self-efficacy and observational learning as key mechanisms (14). Creative arts interventions support mastery experiences in skill building, which elevate confidence in both artistic expression and recovery behaviors (17). Additionally, group-based arts facilitate vicarious learning, as peers model recovery narratives and healthy coping strategies. They also promote verbal persuasion, expressed through feedback in creative sharing contexts (18). Emotional states experienced during artistic “flow” also provide positive affective cues that counter craving-related distress (19).

Narrative Therapy Theory (NTT) posits that individuals are separate from their problems and that dominant, problem-saturated narratives can be externalized and re-authored, paving the way for recovery-oriented identities (15, 16). Arts-based modalities, such as drama, storytelling, visual arts or music, enable youth to externalize addiction, craft alternative preferred stories, and explore aspects of identity suppressed by substance-use narratives (20). This process of externalizing conversations supports re-authoring and increases agency (15). The integration of IPF, SCT, and NTT creates a comprehensive theoretical architecture in which creative arts interventions act both as platforms for narrative meaning-making and self-efficacy skill-building. Recent research reinforces this synergy: arts-based programs have demonstrated effectiveness in enhancing youth knowledge, shifting attitudes, and promoting behavior change in substance-use prevention (21). A 2025 review further confirmed that sustained creative arts engagement correlates with improved adolescent mental health and well-being, reinforcing the importance of experiential, group-based modalities for younger populations (22). Although historical systematic reviews of creative-arts therapies for substance misuse have highlighted limited rigorous evidence, newer scoping and mixed-methods reviews suggest promising pathways for empowerment, peer education, and harm-reduction via participatory arts formats (23). Within this conceptual integration, NTT elucidates how artistic expression helps youth deconstruct addiction-based identities and co-author recovery-focused narratives, while SCT explains how involvement in performing arts builds self-efficacy, peer modelling, and social support, empowering sustained behavioral change (24, 25) Thus, creative arts form an effective mechanism through which young people can access, internalize, and communicate evidence-based insights on SUD while simultaneously reshaping their identity through narrative and reinforcing behavior change through self-efficacy and modelling within a participatory, supportive community.

Together, this integrated framework fosters a holistic, strengths-based approach that provides insights into analyzing both individual behavior and structural influences, thus paving the way for more theoretically grounded, effective, youth-empowering substance abuse interventions.

The Integrated Participatory Framework for Youth-Centred Substance Abuse Intervention (see Table 1) guided the overall research process, ensuring that the study was not only diagnostic but also action-oriented. The initial problem identification and contextual analysis phase informed the selection of study sites and the qualitative interpretive design, as it highlighted the need to combine secondary data analysis with in-depth community engagement to capture both the measurable scope of SUD and its socio-cultural dimensions. This directly aligned with the first objective to gain community perspectives on prevailing substance abuse trends and their effects on the community by providing a narrative foundation for understanding local realities.

**Table 1:** Integrated participatory framework for youth-centered substance abuse intervention.

Phase	Objective	Activities	Expected outcomes	Source
Problem identification and contextual analysis	Define the scope of substance abuse and related challenges in rural communities.	- Analyze datasets (e.g., 79, 81). - Conduct focus groups and interviews with stakeholders.	- Quantified prevalence of substance abuse - Contextual insights into community challenges and perceptions	(72)
Stakeholder engagement	Build a coalition for community-centered and multidisciplinary collaboration.	- Engage with DSD, youth groups, traditional leaders, and local organizations. - Facilitate participatory workshops.	- Stakeholder alignment on goals - Collaborative intervention strategies incorporating local knowledge	(73)
Participatory communication ecology	Disseminate substance abuse data effectively to engage youth and communities.	- Map communication networks. - Develop youth-friendly, culturally relevant materials for dissemination.	- Accessible and engaging content - Increased reach and engagement with youth and community members	(74, 75)
Monitoring, evaluation, and learning (MEL)	Assess the effectiveness and sustainability of interventions.	- Intervene with pre- and post-intervention surveys. - Interview participants qualitatively - Document lessons learned.	- Measured changes in knowledge, attitudes, and behaviors - Improved intervention design for scalability	(76)

The study aims to explore the root causes of substance abuse among the youth in Sekhukhune, and establish the viability of participatory performing arts as a viable approach to engage the youth between the ages of 19–35 with available data and scientific research on SUDs, and associated mental health and behavioral problems. Underlying this broad aim were the following objectives:

- To gain community perspectives on prevailing substance abuse trends and their effects on the community
- To identify socio-structural factors that account for substance abuse among the youth
- To establish suggested ways that can combat substance abuse.

## Research design

This study employed a qualitative interpretive design to explore the nuanced perceptions, lived experiences, and cultural contexts shaping youth SUDs across the Sekhukhune District, including Ga Ngwabe, Praktiseer, Tafelkop, and Ga-Mashashane in Limpopo Province. The qualitative approach was chosen for its strength in facilitating in-depth investigation of complex social phenomena within their natural environments (26), allowing for the construction of rich meaning grounded in participants’ narratives and lived experiences (27). This methodology further aligns with Yin’s (80) case study framework, which emphasizes the integration of multiple data sources to develop a comprehensive, contextualized understanding of the studied phenomenon.

## Context and description of the study area

Sekhukhune District in Limpopo Province represents a uniquely critical context for studying youth substance abuse, due to its convergence of profound socio-economic and structural vulnerabilities. The district experiences chronic poverty, high unemployment, and limited access to education and economic opportunities, which collectively heighten youth susceptibility to substance use and teenage pregnancy (28). The scarcity of recreational and developmental programs further exacerbates youth disengagement, leaving adolescents to adopt risky coping strategies, including drug and alcohol use (28, 29). Compounding these challenges, local governance and social services offer insufficient support, creating a pervasive sense of helplessness despite growing community awareness of youth vulnerabilities (29, 30). Empirical evidence from comparable rural South African settings demonstrates that such intertwined structural, social, and cultural factors significantly shape adverse youth outcomes, underscoring the need for multi-sectoral, community-engaged interventions tailored to local realities (30, 31). Consequently, Sekhukhune provides an ideal setting to examine both the drivers of substance abuse and the potential for locally relevant, evidence-based preventive strategies.

## Sampling

Sites were purposively selected based on documented youth SUDs and demonstrated community readiness for prevention engagement. Following Patton’s (32) recommendation to target information-rich cases, purposive sampling was used to recruit participants with direct experience or knowledge of SUD:

- Youth aged 19–35^1^ years (both in and out of school), *N* = 38
- Traditional leaders and elders, *N* = 9
- Officials from the Department of Social Development, Law enforcement personnel, and educators, *N* = 7
- Representatives from NGOs and community-based organizations involved in youth and public health, *N* = 8.

This approach ensured the inclusion of key informants who offer insider perspectives, vital for designing context-specific interventions.

## Collection methods

### Key informant interviews (KIIs)

Semi-structured KIIs were conducted with community leaders, educators, youth advocates, and service providers. Interview guides explored perceptions of SUD causes, impacts, prevention strategies, and the feasibility of using performing arts for community awareness. Following Brinkmann’s (33) interview methodology, the flexible format allowed probing and adaptation to emergent themes. KIIs addressed Objectives 1 and 3 by eliciting expert and community-level insights into knowledge dissemination and behavior change (34, 35).

### Focus group discussions

Focus group discussions (FGDs) with youth groups (6–10 participants per group) explored collective norms, peer influences, personal experiences, and preferences for creative arts interventions. As Morgan (36) emphasizes, FGDs are valuable for generating interactive data shaped by group dynamics. Discussions supported Objectives 1 and 2 by facilitating peer-to-peer knowledge exchange and collaborative strategy development.

### Non-participant observations

Drawing on Spradley’s (37) ethnographic observation framework, researchers attended community meetings, cultural gatherings, and youth events. Observations captured communication styles, creative expressions, and informal SUD-related discourse, providing contextual depth to KIIs and FGDs. This method advanced Objectives 2 and 3 by identifying cultural platforms that could host arts-based messaging (21).

### Data analysis

All interviews and FGDs were audio-recorded with consent, transcribed verbatim, and translated where necessary. Observation field notes were integrated into the dataset. Data analysis followed Braun and Clarke’s (38) six-phase thematic analysis, supported by NVivo 12 software for systematic coding. Themes were derived from and with project objectives, ensuring analytical coherence between empirical findings and intervention design.

The analysis involved both content and thematic analysis to systematically identify patterns within the data. The process began with repeated reading of the transcripts for data familiarization, which facilitated the development of codes and enabled the researchers to immerse themselves in the data. This was followed by an iterative process of reviewing and refining codes as broad themes emerged. The analysis yielded key themes, including socioeconomic drivers, community responses, barriers to treatment access, and the impact on families and social structures. To enhance the credibility and trustworthiness of the findings, the research team used triangulation, member checking, and peer discussions of emerging results.

### Ethical considerations

Ethical clearance was obtained from the Human Sciences Research Council (HSRC) research ethics committee (Ref: HSRC Research Ethics Committee Protocol No REC 1/24/11/21). Informed consent was obtained from all participants, with parental consent for minors. Anonymity and confidentiality were maintained, and participants could withdraw at any time. Given the sensitivity of SUD discussions, referral information for counselling services was provided.

Rigor in this study was maintained by applying Lincoln and Guba’s (39) foundational criteria of trustworthiness, credibility, transferability, dependability, and confirmability to ensure the findings are robust and trustworthy. Credibility was strengthened through methodological triangulation combining key informant interviews, focus groups, and non-participant observations along with member checking during stakeholder validation workshops to verify interpretations and enhance authenticity (40, 41). This approach aligns with recent evaluations of qualitative research quality, which affirm triangulation, reflexivity, audit trails, and member checks as essential components of credibility (42). Transferability was supported by providing rich, thick descriptions of the socio-cultural and contextual settings, enabling broader applicability of findings in similar environments, as originally proposed by Geertz (43) and restated in more recent qualitative methodology discussions (42, 44, 45). Dependability was ensured through a comprehensive audit trail capturing methodological decisions, coding processes, and analytical reflections consistent with contemporary qualitative rigor recommendations (46). Confirmability was addressed via reflexive journaling and peer debriefing, reducing researcher bias and anchoring findings in participant perspectives. This strategy is corroborated by recent discussions of reflexivity and quality assurance in qualitative research (47, 48). This rigorous approach guarantees that the study’s conclusions are both trustworthy and reflective of lived realities.

## Limitations

This study has several limitations that should be considered when interpreting the findings. First, the manuscript reports on the baseline and formative phase of a larger multi-phase project; consequently, some originally proposed objectives, particularly the evaluation of the intervention’s short-term impact, were not addressed in this phase due to the intervention not yet being implemented. This limits the ability to assess the effectiveness of the arts-based and community engagement strategies discussed. Second, the study relies exclusively on qualitative methods, which provide rich contextual insights but do not offer quantifiable measures of prevalence or intervention outcomes. While this approach was appropriate for exploring complex socio-cultural dynamics, it limits generalized findings and the capacity to demonstrate causal relationships. Third, data were collected from purposively selected communities within the Sekhukhune district, which may limit transferability to other districts or provinces with differing socio-economic or cultural contexts. Finally, the sensitive nature of substance use may have led to underreporting or social desirability bias among participants despite rigorous ethical safeguards and rapport-building strategies. Thus, future research should incorporate mixed-methods designs with longitudinal follow-up to evaluate intervention outcomes and expand geographic scope to enhance applicable findings across diverse settings. A methods matrix was developed to ensure methodological rigor and direct alignment with the study’s three objectives: (1) to gain community perspectives on prevailing substance abuse trends and their effects on the community; (2) to identify socio-structural factors that account for substance abuse among the youth; and (3) to establish suggested ways that can combat substance abuse.

## Findings

The findings from the study reveal a complex interplay of social, economic, and environmental factors – peer influence, institutional gaps, such as the limited role of religious institutions, distrust in law enforcement and structural deficits, including limited recreational and skills-based opportunities shaping youth SUDs in the Sekhukhune District. In particular, the socio-economic context is one of the key drivers of substance abuse in the area, further exacerbated by other social and cultural contextual factors, as outlined in the findings below:

*Socioeconomic vulnerability*: The data reflect that socioeconomic vulnerabilities, such as high unemployment, generational poverty, and limited access to education are perceived to be the root causes and key drivers of substance abuse. Participants articulated how these structural hardships foster feelings of despair and create fertile ground for substance use as a coping mechanism. This insight underscores the importance of enhancing young people’s understanding of the broader systemic factors influencing substance abuse. Understanding these systemic drivers is critical for designing interventions that address the root causes, rather than only the symptoms of substance abuse (28, 29).

*Peer pressure*: Peer pressure and social influence were also recurrent as drivers. The youth participants described how desires for social acceptance and belonging often lead to experimentation with drugs and alcohol. As Bandura (13) explains, individuals often model the behaviors of peers perceived as influential or rewarding (13), thus socially learning these behaviors. This highlights the need to capacitate youth with critical skills for navigating social dynamics and self-efficacy.

*Distrust in law enforcement*: Widespread distrust in policing, often perceived as punitive or procedurally unjust, discourages youth from engaging with formal support systems. As a result, adolescents rely more on informal networks, which may inadvertently reinforce harmful behaviors (30).

*Institutional gaps:* Religious institutions, while culturally trusted, were reported to play a limited role in the community as they are underutilized and unevenly prepared to provide structured support, mentorship, or referrals for substance abuse prevention. This represents a missed opportunity, as faith-based organizations could play a pivotal role in fostering resilience and pro-help-seeking norms (31).

### Systemic and structural drivers

The findings also exposed a significant lack of recreational infrastructure and skills-based education, leaving young people without safe, engaging outlets for their energy and aspirations. This deficit contributes to boredom and vulnerability to substance use, reinforcing the rationale for creative arts-based interventions as culturally resonant platforms for raising awareness and behavioral change.

The data also illuminated community and institutional challenges, including insufficient support from local organizations, limited engagement from spiritual institutions, and pervasive distrust toward law enforcement. These barriers point to the critical need for participatory, culturally grounded intervention design and stakeholder collaboration to ensure sustainability and effectiveness – an overarching principle that connects all project objectives.

This data highlights the physical, mental, and social consequences of substance abuse, including health deterioration, increased crime, and familial disruption. These stark realities emphasize the necessity of targeted knowledge dissemination and behavioral interventions that raise awareness and promote healthier choices.

Collectively, these themes not only deepen understanding of the multifaceted nature of substance abuse in Sekhukhune, but also creatively inform a holistic, youth-centered intervention framework that integrates knowledge enhancement, creative communication, and empowerment for behavioral change.

## Typographical vulnerability

Sekhukhune District reveals a convergence of socio-economic, environmental, and cultural stressors – most notably substance use emerged as the most urgent community concern, a pattern consistent with research across other Limpopo districts and comparable contexts in South Africa and beyond.

*Health consequences "Substances… harm the body because the physical appearance changes… they hardly eat food or even bathe. Alcoholics… are mentally disturbed… People who use drugs suffer the most… they lose weight and lack the energy to perform daily activities."* – Participant C, Tafelkop.

In the Capricorn District, urbanization and the proliferation of informal settlements have similarly increased access to alcohol and illicit drugs, particularly among youth and university students, where peer influence, academic pressure, and the proximity of supply points exacerbate usage (4). Vhembe’s border location mirrors transnational trafficking patterns observed in other African border regions, where porous borders facilitate drug inflow, increasing youth exposure (49). Mopani, like Sekhukhune, experiences the compounded effects of environmental stressors, such as drought on agriculture, employment, and youth migration, with evidence from Ethiopia and Kenya also showing that climate-induced livelihood losses can accelerate substance abuse as a maladaptive coping mechanism (50). The mining-linked school dropout and drug use pattern in Waterberg reflects global trends in mining communities, where extractive economies often disrupt education continuity, reduce youth aspirations, and increase engagement in risky behaviors (51). The lack of recreational infrastructure as a risk factor for youth substance use has also been documented in rural provinces, such as the Eastern Cape and KwaZulu-Natal, and internationally in underserved rural areas of Australia and Canada, where the absence of safe youth spaces correlates with higher rates of alcohol and drug misuse (52).

*Substance types Nyaope… marijuana… even pills which people crush and smoke through the nose. Others smoke cigarettes and glue. The root of the problem is within the household. Parents must be helped through counselling*.” (Participant, B Praktiseer)

Mining-related educational disruptions exacerbate school disengagement, a known precursor to substance abuse, and this pathway is well-supported by empirical studies. Lee and Vandell (53) found that adolescents who spent increased unsupervised out-of-school time were more likely to use substances, underscoring disengagement as a risk factor (53). Lee and Henry (54) demonstrated that strong school engagement in early adolescence significantly reduced alcohol and cannabis use into later teenage years (54). In South Africa, school connectedness has been found to inversely correlate to substance use and other risky behaviors among learners in Durban, particularly in mining-affected areas. Lower levels of school bonding were associated with higher rates of substance use. Supporting this, longitudinal work by Henry et al. (55) found that indicators of school disengagement, such as poor attendance and failure in core subjects predicted increased risk of problematic drug and alcohol use into early adulthood (55). Collectively, these studies affirm that mining-related disruptions to schooling contribute to disengagement, thereby heightening learners’ vulnerability to substance abuse.

*Drug knowledge: "What I know about drugs is that they are harmful substances. Once you smoke drugs, you change completely from the person you used to be … Those who drink alcohol lose morals and consciousness; in the end, they become thieves*." – Participant A, Ngwabe

Overall, the drivers identified in Sekhukhune – peer pressure, economic marginalization, inadequate youth programming, and environmental stressors – align with a broader body of evidence from South Africa and other low-resource settings. This consistency reinforces the urgent need for integrated, community-driven interventions that address both structural determinants and individual-level vulnerabilities.

### Pervasive types of substances

Participants described widespread use of marijuana, nyaope (whoonga), alcohol, inhalants, such as glue and snuff, and prescription pills repurposed for recreational use. This reflects the diversity of substances and the extent to which this a deeply entrenched concern for the community.

### Perceived root causes

Family instability, economic hardship, and inadequate parental guidance emerged as consistent drivers of substance abuse. These patterns were reinforced by observations at community gatherings and youth performances, reflecting broader social and economic challenges in the area.

### Physical and mental health impacts

Participants – particularly in FGDs and during informal exchanges at youth events – described serious health consequences, including weight loss, malnutrition, poor hygiene, and declining mental health. These observations underscore the compounding effects of substance abuse on physical and psychological wellbeing.

### Links to crime

Insights from traditional leaders, law enforcement, and community members revealed a strong association between drug use and theft, driven largely by the need to fund substance purchases.

*Drivers of substance purchases ""People turn to be thieves in the community because they use drugs, and they need money to buy drugs every day."* Participant C, Tafelkop.

Overall, the drivers identified that family instability, economic hardship, inadequate parental guidance, deteriorating health, and the link to criminal activity are consistent with wider evidence from South Africa and other low-resource settings. These findings reinforce the urgency for integrated, community-driven interventions that address both the structural determinants and the individual vulnerabilities underpinning substance abuse.

Family instability, economic hardship, and inadequate parental guidance were consistently identified as drivers of substance abuse. These themes were echoed during observations at community gatherings and youth performances. A young female participant remarked:

Observations and KIIs with traditional leaders and law enforcement indicated a strong link between drug use and theft. An senior citizen male participant confirmed:

In FGDs and youth forums, in all the study sites (Ga Ngwabe, Praktiseer, Tafelkop, and Ga-Mashashane) participants disclosed the risky practice, known as “Bluetooth^2^”– sharing blood between drug users, reflecting a lack of awareness of severe health risks, including HIV transmission.

### Community and institutional gaps

Across data collection methods, participants criticized local churches for prioritizing economic activities over direct support for substance abusers and called for greater spiritual and moral leadership.

### Access points for substances

The Department of Social Development and community stakeholders identified schools, local dealers, taverns, and urban migration as major sources of drug and alcohol access. These observations were corroborated during field visits to hotspot areas.

### Psychosocial and economic impacts on users

Participants repeatedly mentioned loss of self-esteem, family breakdown, health complications, such as HIV, and financial instability, as common consequences of addiction. These patterns were consistent across FGDs, KIIs, and field observations.

### Challenges for civil society and communities

Civil society actors reported persistent barriers, including lack of funding, weak referral systems, safety risks for outreach workers, and community distrust of law enforcement – issues that were also raised informally at youth events and during community dialogues.

Collectively, the consistency of these findings across FGDs, KIIs, observations, and youth event engagements underscores the entrenched nature of substance abuse in Sekhukhune. The convergence of voices across different spaces and stakeholder groups strengthens the validity of the findings and signals an urgent need for coordinated multi-sectoral interventions.

## Discussion

This study set out to examine the root causes, patterns, and consequences of SUDs among youth in the Sekhukhune District of Limpopo, South Africa, with the overarching aim of strengthening young people’s understanding of SUD and promoting creative, participatory prevention strategies. The findings, drawn from key informant interviews (KIIs), focus group discussions (FGDs), observations, and youth event engagements, paint a detailed picture of how structural deprivation, social dynamics, environmental stressors, and institutional weaknesses converge to fuel substance abuse. The consistency of these themes across different data collection methods not only validates their significance but also provides a robust baseline for intervention design.

The first objective, to enhance young people’s knowledge of SUD, was addressed by documenting the range of substances in use, from marijuana and nyaope to inhalants and misused prescription pills, alongside their physical, mental, and social harms. The personal accounts gathered bring lived experiences into sharp relief, offering compelling narratives that can be integrated into educational materials for youth-led awareness campaigns. The second objective, to encourage performing arts as a creative mechanism for knowledge-sharing, is reinforced by the finding that Sekhukhune has a marked shortage of safe, structured recreational and cultural spaces. This gap underscores the potential of arts-based interventions to serve as both an outlet for self-expression and a platform for disseminating prevention messages in ways that resonate with local culture. The third objective, to build youth capacity to access, interpret, and use research-based information, is highlighted by practices, such as “Bluetooth,” which reflect dangerous misconceptions about drug use. Addressing such myths requires empowering young people with accurate, evidence-based knowledge to make informed choices and challenge harmful peer norms. Finally, the fourth objective, to evaluate the short-term impact of interventions is supported by the uniformity of themes across KIIs, FGDs, observations, and youth events. Similar narratives emerged about the prevalence of substance abuse, its root causes, and its consequences. Drug abuse as the most pressing concern, was raised consistently with participants listing multiple substances “Nyaope … marijuana … even pills, which people crush and smoke through the nose,” a pattern echoed by key informants, including local health workers, who confirmed that “these substances are easily accessible in schools, taverns, and through known community dealers.” Observations at youth events corroborated these accounts, with organizers reporting concerns about intoxicated youth attending or loitering near venues.

The patterns observed in Sekhukhune are not isolated. Similar to rural areas in KwaZulu-Natal and the Eastern Cape, the interplay between generational poverty and unemployment here drives psychosocial stress and fosters substance use as an escape mechanism (56, 57). Internationally, research in low-income urban settlements in Brazil and rural Appalachia in the USA shows comparable cycles of deprivation and drug use (58).

Peer pressure and social influence emerged as powerful drivers of experimentation, echoing evidence from Kenya, Botswana, and Namibia, where social acceptance and identity formation during adolescence significantly shape drug-use trajectories (49, 59). These findings align with Social Cognitive Theory (14), which highlights the influence of role models and peer reinforcement in shaping behavior. The paradox of mining investment providing income while driving school dropouts and increasing exposure to high-risk behaviors has parallels in Zambia’s Copperbelt Province and mining towns in the Democratic Republic of Congo, where similar educational disruptions and social vulnerabilities have been observed (60).

Adolescent substance abuse in Sekhukhune reflects the intersection of environmental stressors, institutional shortcomings, and socio-economic precarity (28). The absence of safe recreational facilities and youth-oriented spaces mirrors evidence from Eswatini, Malawi, and Lesotho, as well as from rural Australia and Canada, where young people in under-resourced communities show higher levels of alcohol and drug misuse (61, 62). These dynamics are compounded by drought and climate variability, which destabilize agricultural livelihoods, as similarly documented in Ethiopia and Zimbabwe, where climate shocks trigger migration, unemployment, and substance use. In Sekhukhune, the convergence of these stressors has translated into malnutrition, family disintegration, and crime, with the emergence of the “Bluetooth” practice of blood-sharing for intoxication underscoring how youth develop hazardous coping mechanisms when structural support is absent (63).

Institutional and community gaps intensify these risks. Limited treatment capacity, under-utilization of religious institutions, and distrust in policing create barriers that shape resilience and help-seeking behaviors. Evidence shows that resource shortages and long waiting times discourage care-seeking, leaving adolescents demoralized and disengaged (63, 64). Churches, often the first point of contact, remain constrained by weak referral systems, uneven preparedness, and workforce shortages, undermining their potential to destigmatize substance use and promote pro-help-seeking norms (65).

Regional and international experiences demonstrate that these gaps are not inevitable but reflect missed opportunities for integrated responses. Studies in South Africa, Mozambique, and Tanzania show that fragmented, under-resourced systems consistently weaken prevention and recovery outcomes (66, 67). When contrasting African community-based models, those that connect youth-friendly clinics, trained religious leaders, and police–health collaborations have demonstrated improved service uptake, reduced stigma, and stronger community resilience (68). These findings highlight the fact that resilience can be strengthened through systemic coordination, which recognizes the complementary roles of health, faith, and justice sectors.

The “Bluetooth” phenomenon in Sekhukhune epitomizes the consequences of these systemic failures. Emerging in a context of scarce resources (63), misinformation circulating in peer networks, and inequalities tied to a mining-dependent economy (69), it illustrates how adolescents innovate dangerous alternatives when safe options are unavailable. Unlike high-income settings, where robust public health infrastructures enable evidence-based harm reduction, such as supervised consumption sites and naloxone distribution (70–73). Sekhukhune’s youth operate in contexts where prevention and support are fragmented. This underscores the need for locally grounded strategies that expand recreational opportunities, strengthen treatment systems, mobilize trusted religious institutions, and embed culturally sensitive multi-sectoral approaches that go beyond reactive responses to build resilience and reduce long-term vulnerability.

By situating substance abuse within Sekhukhune’s unique socio-economic, environmental, and cultural realities, this study contributes a nuanced, locally grounded evidence base for intervention design. Its strength lies in the triangulation of consistent findings across multiple qualitative methods and settings, enhancing both credibility and transferability. Importantly, the documentation of emerging high-risk behaviors, such as “Bluetooth,” adds to the regional and global harm reduction evidence base. The findings reveal that substance abuse in Sekhukhune is the product of deep-rooted structural inequalities, environmental pressures, and social disconnection. While the drivers and consequences align with patterns documented in other South African provinces, SADC countries, and global low-resource settings, local manifestations, such as mining-linked educational disruptions and the “Bluetooth” practice, require context-specific solutions. By grounding its insights in lived experiences and aligning them with broader empirical evidence, the study successfully meets its objectives and main aim, offering a compelling foundation for participatory, culturally resonant, and multi-sectoral strategies to address SUD among youth.

This study acknowledges the fact that a sound understanding of the socio-economic and structural inequalities that drive substance abuse trends in Sekhukhune requires a comprehensive mixed-methods research design, which this study does not take. Such a design will yield robust data on the pre- and post-evaluation of implemented interventions, enabling the quantification of outcomes. It is therefore recommended that this research be scaled up into more in-depth research to inform future interventions.

## Conclusion

This study reveals that substance abuse in Sekhukhune District arises from a complex interplay of social, economic, and environmental factors. Persistent poverty, high unemployment, peer pressure, scarce recreational facilities, and recurrent drought collectively heighten youth vulnerability. While mining offers short-term economic benefits, it disrupts education and exacerbates risky behaviors. Triangulated data from key informant interviews, focus groups, observations, and youth events confirm these challenges are systemic, reflecting deep structural inequities, institutional gaps, and insufficient community coordination. The emergence of hazardous practices, such as “Bluetooth,” highlights critical health and harm reduction needs. Effectively addressing substance abuse requires a multi-layered, integrated strategy encompassing economic development, culturally sensitive prevention, institutional reform, and family support. Substance use should not be viewed as an isolated behavioral issue, but rather as a manifestation of deeper structural failures. Addressing it effectively demands simultaneous focus on economic empowerment, educational retention, community engagement, and the strengthening of health systems.

## Recommendations

To translate these insights into impactful action, we propose the following:

Adopt school-based prevention, create safe recreational spaces, and engage faith-based institutions, while tailoring strategies to mining-linked socio-economic and cultural contexts to ensure both local relevance and broader transferability.

Strengthen collaboration among traditional leaders, community organizations, and local government to develop culturally relevant substance abuse prevention and rehabilitation programs. Utilizing creative arts and participatory approaches will engage youth and reshape social norms around drug use through community-centered prevention and rehabilitation centers.

Establish and expand school- and workplace-based counselling and support services focused on early identification and referral. Complement these with structured youth programs offering recreational, vocational, and mentorship opportunities to provide positive alternatives to substance use through Institutional Capacity and Early Intervention programs.

Enforce stricter regulations on substance distribution near schools and workplaces, coupled with community-engaged policing to rebuild trust. Support economic inclusion initiatives, such as youth entrepreneurship grants and community benefit schemes linked to mining, to alleviate poverty-driven vulnerabilities through policy reform and economic empowerment in vulnerable communities.

Advance Family Strengthening and Harm Reduction through developing family-focused counselling programs that address intergenerational poverty and enhance caregiving capacities. Implement harm reduction strategies, including targeted education on risky practices, such as “Bluetooth,” routine health screenings, and expanded access to rehabilitation services.

This study underscores the urgent need for integrated, culturally grounded, and multi-sectoral interventions to disrupt the cycle of substance abuse in Sekhukhune. By addressing structural determinants alongside individual and community-level factors, the recommendations aim to empower youth toward healthier, more productive lives and foster resilient communities capable of sustaining long-term wellbeing. This approach not only aligns with findings across South Africa and the SADC region, but also contributes valuable empirical evidence supporting the design of effective, context-specific substance abuse interventions in similarly affected low-resource settings globally.

## Data Availability

The original contributions presented in the study are included in the article/supplementary material, further inquiries can be directed to the corresponding author.

## Footnotes

1 The age range 19–35 was chosen to capture a broader spectrum of young adults who are still navigating critical transitional life stages, including education completion, employment entry, and family formation. This extended range reflects the local socio-economic realities in Sekhukhune, where challenges such as prolonged unemployment and delayed independence often extend youth vulnerabilities beyond the conventional 15–24 age bracket [77, 78]. [Note: The African Youth Charter is a policy document developed by the African Union Commission.]

2 In Limpopo Province, “*Bluetooth*” refers to the dangerous practice where a person high on drugs, often nyaope, withdraws their own blood and injects it into another person to induce a similar high. This practice carries severe health risks, including HIV and hepatitis transmission, sepsis, and unpredictable drug dosing, and reflects extreme poverty and unsafe injection behaviours among drug-using networks.
